# Modified α,α′-trehalose and d-glucose: green monomers for the synthesis of vinyl copolymers

**DOI:** 10.1098/rsos.171313

**Published:** 2018-05-23

**Authors:** A. Papacchini, M. R. Telaretti Leggieri, L. Zucchini, M. A. Ortenzi, F. Ridi, D. Giomi, A. Salvini

**Affiliations:** 1 Dipartimento di Chimica ‘Ugo Schiff’, Università degli Studi di Firenze, Via della Lastruccia 3/13, Sesto Fiorentino (FI), 50019, Italy; 2 CRC Materiali Polimerici (LaMPo), Dipartimento di Chimica, Via Golgi 19, Milano (MI), 20133, Italy; 3 Consorzio per lo Sviluppo dei Sistemi a Grande Interfase (CSGI), Via della Lastruccia 3, Sesto Fiorentino (FI), 50019, Italy

**Keywords:** trehalose, glucose, vinyl copolymers, renewable resources, green monomers

## Abstract

Allyl saccharide/vinyl copolymers were synthesized using renewable feedstocks (α,α′-trehalose and d-glucose) to obtain ‘green monomers’. Properly designed synthetic procedures were used to obtain copolymers with high purity and without protection/deprotection steps in agreement with the principles of green chemistry and industrial sustainability. The use of saccharide derivatives as monomers allowed products to be obtained that showed high affinity and compatibility for the cellulosic substrates, like paper or wood, and that were suitable for applications like adhesion or consolidation in the field of cultural heritage. All reaction products were characterized by FT-IR and NMR spectroscopies and SEC analyses, while thermal properties were evaluated by DSC analyses.

## 1. Introduction

In the current scenario of dwindling fossil resources and growing environmental concerns connected to the use of fossil fuels, the interest of academia and industries focuses on renewable resources as an appealing alternative to produce energy, fuels and chemicals. Concerning the production of chemicals and in particular polymers, recently an increase in the use of the so-called ‘green monomers’ was observed. Some of them are analogous to widely used petroleum-based monomers (e.g. ethylene), some others are used to synthesize products which simulate the performances of existing petroleum-based polymers and, finally, some are used to synthesize original materials with novel properties and applications [1].

Among the possible green monomers, carbohydrates are of great interest because they are inexpensive and readily available. Furthermore, they are harmless to humans, have low environmental impact and present considerable stereochemical variety. Several examples of the use of carbohydrate-based monomers can be found in the literature. The main strategies are the incorporation of saccharide-based units in the main chain of the polymer, like epoxy resins [2], polyesters, polyamides, polyurethanes and polyureas [3], or the synthesis of polymers having carbohydrate units as pendant groups [4–6].

Carbohydrates are generally derived from biomass, especially lignocellulosic biomass [7]. In particular, the production of d-glucose is one of the most studied processes because this molecule is a versatile precursor to valuable chemicals. d-Glucose can be obtained from lignocellulosic biomass through acid-based or enzymatic hydrolysis of cellulose, or from starch and other natural glucosides. This saccharide is mainly exploited for the production of ethanol, which is nowadays one of the leading actors in the market of biomass-derived fuel [8–10]. Nevertheless, other valuable chemicals like hydroxymethylfurfural, 1,3-propanediol, lactic acid, succinic acid, levulinic acid and sorbitol can be obtained from d-glucose using different techniques (e.g. dehydration, fermentation, hydrolytic hydrogenation and acid treatments) [11]. Among them, methyl d-glucosides deserve particular attention because they are stable even under severe conditions due to the protection of the anomeric position. The α- and β-methyl-d-glucosides are used as raw materials for paints, cosmetics, surfactants and detergents [12–16]. They can be obtained directly from cellulose in supercritical methanol [17] through alcoholysis reactions in methanol using heteropolyacid catalysts [18,19], or from d-glucose using sulfonated-type cation exchange resins [20,21]. As green monomers, the methyl glucoside derivatives are studied for the synthesis of glycopolymers for biological applications [22]. Moreover, cornstarch-derived methyl-d-glucosides have been tested by Dunn *et al.* [23] as multi-functional monomers (MFMs) after their conversion to polymerizable derivatives (i.e. allyl ethers, acrylates and methacrylates). These renewable MFMs were copolymerized with methyl methacrylate or graft-copolymerized with polypropylene and they gave good results in terms of improvement of glass transition temperatures (*T*_g_), torsional and elastic modulus. Concerning the wood industry, the methyl d-glucosides have been tested as extenders for phenol-formaldehyde resins obtaining satisfactory results [24–27].

Another interesting example of a saccharide suitable for the synthesis of green monomers is α,α′-trehalose, which is widely found in bacteria, fungi, plants and invertebrates where it plays different roles (e.g. source of carbon and/or energy, structural component of bacterial cell walls, stabilizer and protectant of proteins and membranes against environmental stresses, sensing compound and/or growth regulator) [28,29]. The α,α′-trehalose is a nonreducing disaccharide with a symmetrical structure and a higher stability against acids and heat than other reducing disaccharides. Although this molecule shows unique properties, studies on the synthesis of α,α′-trehalose-based polymers have only increased in the literature in recent years. The reason for this is related to the production costs, which significantly decreased after the discovery of a new effective and inexpensive method of enzymatic industrial synthesis from starch [30,31]. Recent studies include the preparation of α,α′-trehalose-based polymer networks through vinyl benzyl etherification and subsequent thermal curing reactions [32] or through allyl etherification and subsequent thiol-ene photopolymerization [33]. Moreover, linear polymers were synthesized using polyaddition of diamino-type α,α′-trehalose with diisocyanates [34], enzymatic or chemoenzymatic reactions [35,36], acetalization reactions with dialdehydes [37,38], hydrosilylation [39], Diels–Alder [40] and azide–alkyne Huisgen reactions.

However, due to the polyfunctionality of the starting products, the synthetic methods generally used to obtain polymers from carbohydrates are not always in line with the 12 principles of Green Chemistry developed by Anastas & Warner [41] and with the requirement of industrial sustainability. These methods include complex synthetic procedures, like protection/deprotection steps, that are necessary to obtain products with specific properties, but whose use is not essential when the final polymers are meant to be used in applications like the adhesion or the consolidation for conservation purposes.

Concerning this latter field of application, the choice of saccharides as green monomers presents further advantages, besides the fact that their use is independent of fossil fuels. In fact, the presence of saccharides in the structure of a polymer enables an increase in its compatibility and affinity for works of art made of the same chemical species that constitutes the lignocellulosic biomass (i.e. wood and paper). In the literature, there are several examples of the use of saccharides [42–44], natural polysaccharides or their derivatives [45–49], or of oligomers based on natural molecules with a polar structure similar to the linear form of saccharides [50,51] for the consolidation of paper and wooden works of art. Furthermore, for their use on cultural heritage materials other important requirements must be satisfied, such as the lack of impurities and the stability of the products selected for the restoration interventions.

In the present work new synthetic biopolymers (according to the definition given by the standard CEN/TR 15932 [52]) were synthesized using renewable feedstocks (α,α′-trehalose and d-glucose) and properly designed synthetic procedures, obtaining final vinyl copolymers that have high purity, stability and affinity for the cellulosic substrates, like paper or wood.

## 2. Materials and methods

### 2.1. Materials

α-d-Glucose, α,α′-trehalose, allyl bromide, Amberlite IR-120H resin, hydrochloric acid, ethanol, methanol, vinyl acetate, D_2_O, CD_3_OD and CDCl_3_ were purchased from Sigma Aldrich. Potassium hydroxide was purchased from Carlo Erba. Azobisisobutyronitrile (AIBN) was purchased from Fluka. Acetone was purchased from VWR International. All the chemicals were reagent grade and were used without further purification.

Amberlite IR-120H resin (250 mg) was activated by washing with methanol (3 × 10 min, 1.25 ml each time) and standing overnight in methanol (1.25 ml).

### 2.2. Methods

#### 2.2.1. Synthesis of methyl d-glucopyranoside (MG)

In a Sovirel® tube α-d-glucose (1.7 mmol) and methanol (6.0 ml) were added to 300.0 mg of activated Amberlite IR-120H resin and the reaction mixture was allowed to react at 95°C for 24 h under gentle stirring. After cooling to room temperature, the mixture was filtered on a Büchner funnel and then the solution was distilled under reduced pressure. The white solid was dried in vacuum at room temperature (309.9 mg, 96% yield). ^1^H-NMR (D_2_O, 400 MHz, ppm): 3.27 (1H, t, H_2_^α^); from 3.36 to 3.41 (2H, m, H_5_^α^, H_5_^β^); 3.43 (3H, s, –O-C**H_3_**^α^); from 3.45 to 3.52 (2H, m, H_2_^β^, H_3_^β^); 3.56 (1H, m, H_4_^α^); 3.58 (3H, s, –O-C**H_3_**^β^); from 3.63 to 3.68 (2H, m, H_3_^α^, H_4_^β^); from 3.70 to 3.96 (4H, m, H_6_^α^, H_6_^β^); 4.39 (1H, d, H_1_^β^); 4.82 (1H, d, H_1_^α^). ^13^C-NMR (D_2_O, 100 MHz, ppm): 55.0 (–O-**C**H_3_^α^); 57.1 (–O-**C**H_3_^β^); 60.5 (C_6_^α^); 60.7 (C_6_^β^); 69.5 (C_5_^α^); 69.6 (C_5_^β^); 71.2 (C_4_^α^); 71.5 (C_3_^α^); 73.0 (C_2_^α^, C_4_^β^); 75.7 (C_3_^β^); 75.8 (C_2_^β^); 99.2 (C_1_^α^); 103.2 (C_1_^β^). FT-IR (KBr pellets): peaks at 3383 (s, O–H stretching); 2912 (m, –C–H stretching); 1144, 1102, 1074, 1047, 1029 (s, C–OH stretching, C–O–C stretching) cm^−1^.

#### 2.2.2. Synthesis of the monomers

##### 2.2.2.1. Synthesis of allyl α,α′-trehalose (ATR)

In a Sovirel® tube an aqueous solution of KOH (6.0 ml, 2.1 M) was added under nitrogen atmosphere to α,α′-trehalose (1.6 mmol) and the mixture was heated at 60°C for 1 h. After cooling to room temperature, allyl bromide (0.8 ml, 9.6 mmol) was added under nitrogen atmosphere and the reaction mixture was allowed to react at 60°C for 48 h under vigorous stirring. After cooling to room temperature, the pH was adjusted to neutrality using HCl (2 N) and finally the solvent and the residual allyl bromide were distilled at reduced pressure. The solid residue was extracted in ethanol to separate the product from salts. The alcoholic phase was distilled at reduced pressure and a white solid was obtained (673.0 mg, DS = 1.3, 97% yield). ^1^H-NMR (D_2_O, 400 MHz, ppm): from 3.40 to 3.97 (12H, m, H_2_–H_6_, H_2_′–H_6_′); from 4.09 to 4.37 (2H, m, –C**H_2_**–CH=CH_2_ allyl group); 5.21 (2H, m, H_1_, H_1_′); from 5.28 to 5.41 (2H, m, –CH_2_–CH=C**H_2_** allyl group); 5.97 (1H, m, –CH_2_–C**H**=CH_2_ allyl group). ^13^C-NMR (D_2_O, 100 MHz, ppm): 60.0, 60.2, 60.5 (C_6_, C_6_′); 68.4 (C_6_ functionalized, C_6_′ functionalized); 69.4, 69.6, 69.8 (C_4_, C_4_′); 70.6, 70.9, 71.1 (C_2_, C_2_′); from 71.6 to 72.4 (C_5_, C_5_′, C_3_, C_3_′); 73.9 (–**C**H_2_–CH=CH_2_ allyl group); 77.4 (C_4_ functionalized, C_4_′ functionalized); 78.0 (C_2_ functionalized, C_2_′ functionalized); 91.1, 93.1, 93.2, 93.6 (C_1_, C_1_′); 118.3, 118.5, 118.7, 118.8, 119.2 (–CH_2_–CH=**C**H_2_ allyl group); 133.4, 133.5, 133.7 (–CH_2_–**C**H=CH_2_ allyl group). FT-IR (KBr pellets): peaks at 3390 (s, O–H stretching); 3081, 3017 (w, =C–H stretching); 2933 (m, –C–H stretching); 1645 (m, C=C stretching); 1149, 1105, 1076, 1047, 993 (s, C–OH stretching, C–O–C stretching); 941 (m, =C–H out of plane bending) cm^−1^.

##### 2.2.2.2. Synthesis of allyl methyl d-glucopyranoside (AMG)

In a Sovirel® tube an aqueous solution of KOH (1.0 ml, 3.9 M) was added under nitrogen atmosphere to methyl d-glucopyranoside (1.1 mmol) and the mixture was heated at 70°C for 1 h. After cooling to room temperature, allyl bromide (0.28 ml, 3.3 mmol) was added under nitrogen atmosphere and the reaction mixture was allowed to react at 70°C for 48 h under vigorous stirring. After cooling to room temperature, the pH was adjusted to neutrality using HCl (2N) and the solvent and the residual allyl bromide were distilled at reduced pressure. The solid residue was extracted in ethanol to separate the product from salts. The alcoholic phase was distilled at reduced pressure and a pale yellow solid was obtained (239.4 mg, DS = 1.1, 90% yield). ^1^H-NMR (D_2_O, 400 MHz, ppm): from 3.18 to 3.36 (1H, m, H_2_^α^); 3.46 (3H, s, –O-C**H_3_**^α^); 3.62 (3H, s, –O-C**H_3_**^β^); from 3.37 to 3.94 (9H, m, H_3_^α^–H_6_^α^, H_2_^β^–H_6_^β^); from 4.13 to 4.38 (2H, m, –C**H_2_**–CH=CH_2_ allyl group); from 4.64 to 4.74 (1H, m, H_1_^β^); from 4.83 to 4.86, from 5.00 to 5.03, from 5.24 to 5.28 (1H, m, H_1_^α^); from 5.30 to 5.42 (2H, m, –CH_2_–CH=C**H_2_** allyl group); 6.01 (1H, m, –CH_2_–C**H**=CH_2_ allyl group). ^13^C-NMR (D_2_O, 100 MHz, ppm): 54.7 (–O-**C**H_3_^α^); 57.1 (–O-**C**H_3_^β^); 60.3, 60.5, 60.7 (C_6_^α^, C_6_^β^); 68.4, 68.6, 68.8 (C_6_^α^ functionalized, C_6_^β^ functionalized); 69.6, 69.8 (C_5_^α^, C_5_^β^); 71.2, 71.4 (C_4_^α^); 71.5 (C_3_^α^); 71.7, 72.0, 73.7 (–**C**H_2_–CH=CH_2_ allyl group); 74.0, 74.6 (C_2_^α^, C_4_^β^); 75.7, 75.9 (C_2_^β^, C_3_^β^); 77.4 (C_4_^α^ functionalized, C_4_^β^ functionalized); 78.3 (C_2_^α^ functionalized, C_2_^β^ functionalized); 95.8 (C_1_^α^); 99.2 (C_1_^β^); 118.7 (–CH_2_–CH=**C**H_2_ allyl group); 133.6 (–CH_2_–**C**H=CH_2_ allyl group). FT-IR (KBr pellets): peaks at 3381 (s, O–H stretching); 3080, 3011 (w, =C–H stretching); 2935 (m, –C–H stretching); 1645 (m, C=C stretching); 1146, 1103, 1076, 1051 (s, C–OH stretching, C–O–C stretching); 930 (w, =C–H out of plane bending) cm^−1^.

#### 2.2.3. Synthesis of copolymers with vinyl acetate

##### 2.2.3.1. Synthesis of allyl α,α′-trehalose/vinyl acetate (ATR/VAc) copolymer

(a) In a Sovirel® tube vinyl acetate (6.5 mmol, 0.60 ml) and a solution of allyl α,α′-trehalose in methanol (0.5 mmol in 2.5 ml) were added under nitrogen atmosphere to azobisisobutyronitrile (AIBN) (22.7 mg) and the reaction mixture was allowed to react at 90°C for 6 h under continuous stirring. After cooling to room temperature, the solvent and the residual vinyl acetate were distilled at reduced pressure. The solid residue was extracted in acetone and the soluble fraction was distilled at reduced pressure obtaining a pale orange solid (fraction A + B, 491.4 mg, 65% yield), which was characterized by ^1^H-NMR (CD_3_OD/200 MHz, table 1), ^13^C-NMR (CD_3_OD/50 MHz, table 2), FT-IR (KBr pellets, table 3), SEC and DSC analyses. Finally, the solid was extracted using Milli-Q water and two fractions were obtained: a water-soluble fraction (fraction A, 122.28 mg, 16% yield) and a water-insoluble fraction (fraction B, 368.55 mg, 49% yield). Fractions A and B were characterized by ^1^H-NMR (D_2_O/400 MHz for fraction A and CDCl_3_/400 MHz for fraction B, table 1), ^13^C-NMR (D_2_O/100 MHz for fraction A and CDCl_3_/100 MHz for fraction B, table 2), FT-IR (KBr pellets, table 3), SEC and DSC analyses.

**Table 1.** ^1^H-NMR signals of vinyl copolymers.

		ATR/VAc copolymer			AMG/VAc copolymer			ATR/VOH copolymer		AMG/VOH copolymer	
^1^H-NMR (ppm)		fraction A + B	fraction A	fraction B	fraction A + B	fraction A	fraction B	fraction 1	fraction 2	fraction 1	fraction 2
vinyl acetate unit	CH_2_	1.84	1.96	1.74, 1.83	1.82	1.86	1.71, 1.79				
	CH_3_	2.00, 2.03	2.13, 2.17, 2.23, 2.27	1.97, 2.00, 2.01	1.99, 2.02, 2.04	2.04, 2.07, 2.08, 2.10	1.95, 1.98, 2.00				
	CH	4.87	4.97, 5.06	5.14	4.90	4.92	4.83				
α,α′-trehalose unit	H_1_, H_1_′	5.11	5.23	5.14				5.14, 5.15, 5.16, 5.17	5.18, 5.20		
	H_2_–H_6_, H_2_′–H_6_′	3.40–3.97	3.39–3.97	2.70–4.20				3.39–3.96	3.39–3.92		
methyl d-glucopyranoside unit	H_1_^α^, H_1_^β^				4.66, 4.81	4.60–4.90	4.70			4.34, 4.38, 4.54	4.33, 4.35
	H_2_^α^–H_6_^α^, H_2_^β^–H_6_^β^				3.00–4.00	3.11–3.90	2.90–3.90			3.20–3.88	3.20–3.95
	–OCH_3_^α^				3.40	3.36	3.36			3.33	3.38
	–OCH_3_^β^				3.53	3.51	3.49			3.40	3.53
allyl group	CH_2_=	5.18–5.34	5.30–5.41	5.25	5.08–5.34	5.12, 5.24, 5.33	5.14–5.26	5.24–5.36	5.24–5.39	5.23, 5.28, 5.37	5.22–5.34
	=CH	5.95	6.00	5.88	5.91	5.90	5.88	5.94	5.97	5.96	5.93
	CH_2_	4.03–4.38	4.12–4.45	4.34	3.98–4.40	4.00–4.34	4.03–4.30	4.16–4.34	4.17–4.37	4.15, 4.28	4.01–4.30
vinyl alcohol unit	CH_2_							1.58, 1.60, 1.61, 1.67, 1.68, 1.70	1.61, 1.64, 1.67, 1.71, 1.74	1.60, 1.62, 1.66, 1.69	1.57, 1.59, 1.61, 1.67
	CH							4.01	4.02	4.01	3.99

**Table 2.** ^13^C-NMR signals of vinyl copolymers.

		ATR/VAc copolymer			AMG/VAc copolymer			ATR/VOH copolymer		AMG/VOH copolymer	
^13^C-NMR (ppm)		fraction A + B	fraction A	fraction B	fraction A + B	fraction A	fraction B	fraction 1	fraction 2	fraction 1	fraction 2
vinyl acetate unit	CH_2_	40.0, 40.4, 40.7, 41.1	38.3	38.7, 39.1, 39.9	39.9, 40.3, 40.7, 41.0	38.4, 39.0, 41.0	38.6, 39.0, 39.4, 39.8				
	CH_3_	21.3	20.7	21.0	21.2	20.3, 20.8	20.9, 21.0				
	CH	68.2, 68.6, 68.8	67.8, 68.0	66.0, 66.3, 66.6, 66.9, 67.9	67.8, 68.1, 68.5, 68.8	68.8	66.3, 66.6, 66.9, 67.8				
	CO	172.4	173.3	170.3	172.3	173.6	170.2				
α,α′-trehalose unit	C_1_, C_1_′	95.2	91.3, 93.1, 93.2, 93.3	—				93.1	93.4		
	C_2_–C_5_, C_2_′–C_5_′	69.8–74.9	69.5–72.5	—				69.6, 71.0, 72.1, 72.4	69.9, 71.2, 72.3, 72.7		
	C_6_, C_6_′	62.8	60.2, 60.3, 60.5	—				60.4	60.7		
	C_2_, C_2_′ funct.	—	78.1	—							
	C_4_, C_4_′ funct.	—	77.6	—							
	C_6_, C_6_′ funct.	—	68.5	—							
methyl d-glucopyranoside unit	C_1_^α^, C_1_^β^				99.3, 101.3, 105.5	97.4, 99.4, 103.4	—			97.3, 99.4, 103.4	97.1, 99.2, 103.2
	C_2_^α^–C_5_^α^, C_2_^β^–C_5_^β^ C_2_^α^, C_2_^β^ funct. C_4_^α^, C_4_^β^ funct. C_6_^α^, C_6_^β^ funct.				69.7–81.0	69.4–83.7	68.0–75.0			69.7, 71.4, 71.7, 73.2, 75.9, 76.0	69.5, 71.1, 71.5, 73.0, 75.7, 75.8
	C_6_^α^, C_6_^β^				62.8	62.8	62.8			60.7, 60.9	60.7, 60.5
	–OCH_3_^α^				55.6	55.2	55.2			55.2	54.9
	–OCH_3_^β^				57.3	57.3	57.0			57.3	57.1
allyl group	CH_2_=	116.7, 116.8, 117.3	118.2, 118.5, 118.8, 119.1	—	116.7, 117.2, 117.6	118.5, 118.8	—	—	—	—	118.3, 118.7
	=CH	136.5, 136.8	133.5, 133.6, 133.9	—	136.1, 136.5	134.0, 134.3	—	—	—	—	133.9
	CH_2_	—	71.7	—	72.5	73.2	—	—	—	—	72.2
vinyl alcohol unit	CH_2_							43.3, 43.9, 44.1, 44.5, 44.7	43.6, 44.1, 44.2, 44.6, 44.9	43.6, 44.1, 44.3, 44.7, 44.8	43.3, 43.8, 44.1, 44.5, 44.6, 44.7
	CH							64.6, 66.0, 66.2, 67.6	65.0, 66.3, 66.5, 67.9	64.9, 66.3, 66.5, 67.9	64.6, 65.9, 66.2, 67.6

**Table 3.** FT-IR bands of vinyl copolymers.

	ATR/VAc copolymer			AMG/VAc copolymer			ATR/VOH copolymer		AMG/VOH copolymer	
FT-IR (cm^−1^)	fraction A + B	fraction A	fraction B	fraction A + B	fraction A	fraction B	fraction 1	fraction 2	fraction 1	fraction 2
O–H str.	3435	3381	3460	3420	3383	3495	3355	3360	3399	3394
–C–H str.	2935	2933	2933	2926	2930	2930	2937	2943	2938	2943
C=O str. acetate group	1736	1738	1738	1734	1732	1732	1747	—	1750	1747
C=C str.	1647	1647	1652	1647	1645	1647	1645	1650	1652	1647
CH_3_– δ acetate group	1433, 1375	1425, 1373	1435, 1373	1437, 1373	1456, 1373	1435, 1373	1436	1411	1435	1420
C–O str. acetate group	1244	1247	1244	1244	1253	1242				
C–OH str., C–O–C str.	1146, 1105, 1080, 1043, 1024, 995	1149, 1105, 1078, 1047, 993	1105, 1022, 997	1147, 1103, 1079, 1047, 1026	1148, 1105, 1074, 1047	1105, 1045, 1022	1144, 1104, 1082, 1051, 993	1148, 1102, 1082, 1050, 995	1141, 1080, 1050	1146, 1104, 1079, 1050
=C–H δ out of plane	945	941	945	946	930	947	942	943	920	920

(b) The reaction was repeated using ethanol as solvent: fraction A + B, 4687 mg, 62% yield; fraction A, 149,99 mg, 20% yield, fraction B, 317 52 mg, 42% yield. All fractions were characterized by ^1^H-NMR, ^13^C-NMR and FT-IR.

##### 2.2.3.2. Synthesis of allyl methyl d-glucopyranoside/vinyl acetate (AMG/VAc) copolymer

In a Sovirel® tube vinyl acetate (5.8 mmol, 0.5 ml) and a solution of allyl methyl d-glucopyranoside in methanol (0.9 mmol in 2.5 ml) were added under nitrogen atmosphere to azobisisobutyronitrile (AIBN) (20.3 mg) and the reaction mixture was allowed to react at 90°C for 6 h under continuous stirring. After cooling to room temperature, the solvent and the residual vinyl acetate were distilled at reduced pressure. The solid residue was extracted in acetone and the soluble fraction was distilled at reduced pressure obtaining a yellow-orange solid (fraction A + B, 402.1 mg, 57% yield), which was characterized by ^1^H-NMR (CD_3_OD/200 MHz, table 1), ^13^C-NMR (CD_3_OD/50 MHz, table 2), FT-IR (KBr pellets, table 3), SEC and DSC analyses. Finally, the solid was extracted using Milli-Q water and two fractions were obtained: a water-soluble fraction (fraction A, 80.42 mg, 12% yield) and a water-insoluble fraction (fraction B, 321.68 mg, 46% yield). Fractions A and B were characterized by ^1^H-NMR (D_2_O/200 MHz for fraction A and CDCl_3_/400 MHz for fraction B, table 1), ^13^C-NMR (D_2_O/50 MHz for fraction A and CDCl_3_/100 MHz for fraction B, table 2), FT-IR (KBr pellets, table 3), SEC and DSC analyses.

#### 2.2.4. Synthesis of copolymers with vinyl alcohol

##### 2.2.4.1. Synthesis of allyl α,α′-trehalose/vinyl alcohol (ATR/VOH) copolymer

In a dry two-neck flask equipped with a bubble condenser and an isobaric dropping funnel, anhydrous methanol (3.2 ml) was added to KOH (32.1 mg) under nitrogen atmosphere and the mixture was maintained at room temperature (25°C) until the solid was dissolved. Then, a solution of the allyl α,α′-trehalose/vinyl acetate copolymer (fraction A + B) in anhydrous methanol (8.1 mmol in 4.7 ml) was added using the dropping funnel under nitrogen atmosphere and the mixture was allowed to react at 50°C for 1 h under continuous stirring. The precipitate formed during the reaction was separated from the solution by a centrifugation, dried under vacuum at room temperature (fraction 1, 332.6 mg) and characterized by ^1^H-NMR (D_2_O/400 MHz, table 1), ^13^C-NMR (D_2_O/100 MHz, table 2), FT-IR (KBr pellets, table 3) and DSC analysis. The solution previously recovered after the centrifugation was distilled at reduced pressure and the solid residue (fraction 2, 171.2 mg) was characterized by ^1^H-NMR (D_2_O/200 MHz, table 1), ^13^C-NMR (D_2_O/50 MHz, table 2), FT-IR (KBr pellets, table 3) and DSC analysis.

##### 2.2.4.2. Synthesis of allyl methyl d-glucopyranoside/vinyl alcohol (AMG/VOH) copolymer

In a dry two-neck flask equipped with a bubble condenser and an isobaric dropping funnel, anhydrous methanol (2.3 ml) was added to KOH (22.8 mg) under nitrogen atmosphere and the mixture was maintained at room temperature (25°C) until the solid was dissolved. Then, a solution of allyl methyl d-glucopyranoside/vinyl acetate copolymer in anhydrous methanol (6.1 mmol in 3.3 ml) was added using the dropping funnel under nitrogen atmosphere and the mixture was allowed to react at 50°C for 1 h under continuous stirring. The precipitate formed during the reaction was separated from the solution by a centrifugation, dried under vacuum at room temperature (fraction 1, 232.30 mg) and characterized by ^1^H-NMR (D_2_O/200 MHz, table 1), ^13^C-NMR (D_2_O/50 MHz, table 2), FT-IR (KBr pellets, table 3) and DSC analysis. The solution previously recovered after the centrifugation was distilled at reduced pressure and the solid residue (fraction 2, 426.5 mg) was characterized by ^1^H-NMR (D_2_O/400 MHz, table 1), ^13^C-NMR (D_2_O/100 MHz, table 2), FT-IR (KBr pellets, table 3) and DSC analysis.

### 2.3. Instruments

^1^H-NMR, ^13^C-NMR spectra were recorded with a Varian Mercury Plus 400 spectrometer and a Varian VXR 200 spectrometer working at 399.921 MHz and 199.985 MHz, respectively. The chemical shifts are reported in ppm and referred to TMS as internal standard. Spectra elaboration was performed with the software MestRe-C 4.3.2.0.

FT-IR spectra were recorded with a Shimadzu FT-IR-8400S model and elaborated with the software Shimadzu IRsolution 1.04. Spectra of solid samples were recorded as KBr pellets.

The ICP analysis was performed with a PerkinElmer ICP-OES Spectrometer OPTIMA 2000 DV.

Differential Scanning Calorimetry (DSC) experiments were performed with a DSC Q2000 (TA Instruments) calorimeter. Samples were pre-dried using a vacuum pump and freeze-dried. Then, they were closed in aluminium hermetic pans and analysed from −40°C to 100°C at 10°C min^−1^. Two heating-cooling cycles were acquired and the *T*_g_ of the polymers were measured on the second heating scan.

The size exclusion chromatography (SEC) system was composed of a Waters 1515 Isocratic HPLC pump and a three Phenogel columns set (443-K0, 445-K0, 446) equipped with a Waters 2487 Dual *λ* Absorbance Detector set at 230 nm using 1 ml min^−1^ flow rate and 20 µl as injection volume. Samples were prepared dissolving 10–20 mg of polymer in 1 ml of anhydrous DMF. Before the analysis, the solution was filtered with 0.45 µm filters, therefore the reported results refer only to the polymer present in solution. o-Dichlorobenzene was used as an internal reference (5 µl ml^−1^). The molecular weights were determined using a calibration made with monodisperse polystyrene standards.

## 3. Results and discussion

### 3.1. Synthesis and characterization of the monomers

**Figure 1.**
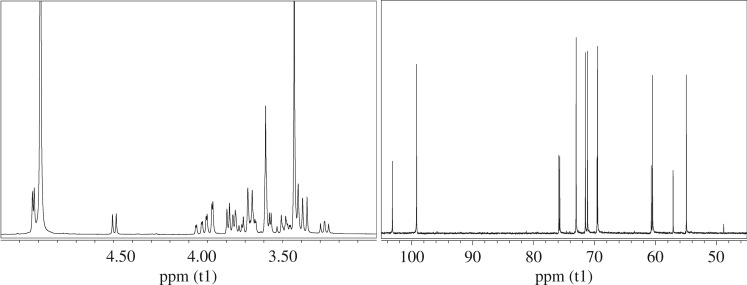
^1^H-NMR and ^13^C-NMR spectra of methyl d-glucopyranoside (MG).

Two different saccharides were chosen as renewable starting materials for the synthesis of the monomers. The α,α′-trehalose is a nonreducing disaccharide and consequently a direct functionalization of the molecule was possible. On the contrary, the α-d-glucose is a reducing saccharide with a highly reactive free anomeric position. Therefore, a reaction in methanol in the presence of an ion exchange resin (i.e. Amberlite IR-120H) as a catalyst was necessary to protect the anomeric position by methylation of the hydroxyl group. The final product was a mixture of α and β methyl d-glucopyranoside (MG) as confirmed by ^1^H-NMR and ^13^C-NMR spectra (figure 1). In particular, the α form was present in a higher amount compared to the β form, with a ratio of 2 : 1, which was calculated using the integral values of the methoxy signals (3.46 ppm, ─O─CH3^α^; 3.62 ppm, ─O─CH_3_^β^) in the ^1^H-NMR spectrum and was consistent with the *anomeric effect*.

**Scheme 1.**
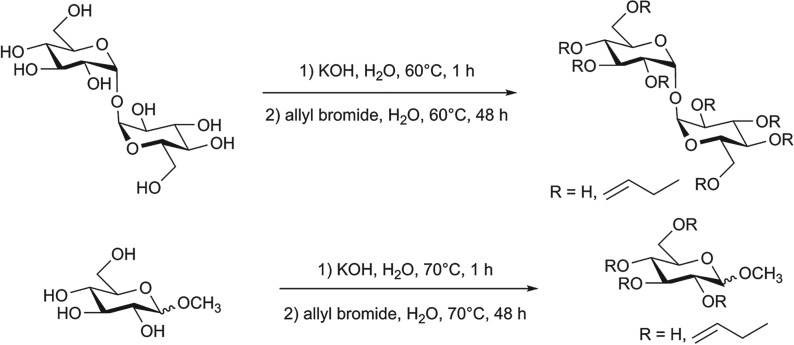
Synthesis of the monomers (ATR and AMG).

The syntheses of the monomers aimed at introducing allyl groups in the structure of the saccharides. Recently, Nagashima *et al.* [33] performed the functionalization of α,α′-trehalose using DMSO as a solvent. In the present research, water was chosen as a ‘greener’ solvent compared to DMSO. The allyl bromide was used as a functionalizing agent because the reactivity of the allyl group can be exploited in the subsequent copolymerizations. The syntheses of the monomers (scheme 1) were performed under nitrogen atmosphere in order to avoid the oxidation of the allyl groups in the presence of oxygen and high temperatures. Strong basic conditions were maintained during the reactions to activate the hydroxyl groups of the saccharides, thus promoting the nucleophilic substitution on allyl bromide. KOH was used for this purpose, also because in this way it was possible to improve the purification of the final products with respect to previous researches [49]. In fact, even if NaOH is generally used in the synthesis of saccharide ethers to activate the hydroxyl groups, sodium salts are more soluble than potassium salts in the solvent used to purify the monomers (i.e. ethanol) [53]. Therefore, sodium salts may be extracted in ethanol together with the monomers in higher quantities compared to potassium salts. ICP elemental analysis showed that the final products contained 3% potassium, which corresponded to 10% of impurities, in the hypothesis that all the potassium was present in the form of KBr (the heaviest among the possible by-products).

Allyl α,α′-trehalose (ATR) and allyl methyl d-glucopyranoside (AMG) were obtained with yields greater than 90% and their characterization was performed by NMR and FT-IR spectroscopies (electronic supplementary material, figures S1–S3). The characteristic signals of the allyl group were present in the NMR spectra of both monomers at similar chemical shifts. In particular, in the ^1^H-NMR spectra the signals were present at about 4.10–4.40 ppm (–C**H_2_**–CH=CH_2_), 5.30–5.40 ppm (–CH_2_–CH=C**H_2_**) and 6.00 ppm (–CH_2_–C**H**=CH_2_), and in the ^13^C-NMR spectra at about 72.0–74.0 ppm (–**C**H_2_–CH=CH_2_), 118.0–119.0 ppm (–CH_2_–CH=**C**H_2_) and 133.5 ppm (–CH_2_–**C**H=CH_2_). Moreover, in all the NMR spectra the signals related to the structures of α,α′-trehalose or methyl d-glucopyranoside were split and broadened with respect to those of the starting saccharides. This result was attributed to the functionalization of hydroxyl groups in different positions. In particular, the presence of some new signals in the ^13^C-NMR spectra of both the monomers at about 68.5 ppm, 77.4 ppm and 78.0 ppm confirmed this hypothesis because they were assigned to three different functionalized carbon atoms (C_6_, C_4_ and C_2_, respectively). Finally, the formation of the desired products was also confirmed by the FT-IR spectroscopy thanks to the presence of the =C–H stretching at 3080, 3011 cm^−1^, the C=C stretching at 1645 cm^−1^ and the =C–H out of plane bending at 940 cm^−1^ in the spectra of both monomers. To evaluate the extent of the functionalization, a parameter that is widely used in cellulose chemistry, namely the degree of substitution (DS), was used. The term ‘degree of substitution’ generally refers to the average number of functional groups on the units of a polymer, but in this research it referred to the average number of functional groups per molecule of saccharide. The DS was calculated using the integral values of the characteristic signals in the ^1^H-NMR spectra. Based on DS calculations, the extent of the monomers' functionalization and their molecular weights were evaluated. The DS for the ATR was calculated using the following formula:

$$\text{DS}=\frac{A\cdot N}{\left(B^{\prime }-2A)+(B^{\prime \prime }-2A\right)}$$

where *A* is the value of the integral of the signal at about 6.00 ppm (–CH_2_–C**H**=CH_2_ allyl group); *B′* is the value of the integral of the signal at 5.30–5.40 ppm (H_1_, H_1_′ α,α′-trehalose and –CH_2_–CH=C**H_2_** allyl group); *B*^″^ is the value of the integral of the signal at 3.40–4.40 ppm (H_2_–H_6_, H_2_′–H_6_′ α,α′-trehalose and –C**H_2_**–CH=CH_2_ allyl group); and *N* is the total number of the protons of the disaccharide giving signals at 5.30–5.40 ppm and at 3.40–4.40 ppm (14 for α,α′-trehalose).

The calculation for AMG was easier because the signals of H_2_–H_6_ and –O–C**H_3_** of both α and β forms of the MG (9 protons) had chemical shifts between 3.18 ppm and 4.00 ppm in the ^1^H-NMR spectrum. Therefore, by setting the integral of this signal equal to 9 using the software MestRe-C 4.3.2.0, the value obtained for the integral of the signal at about 6.00 ppm (–CH_2_–C**H**=CH_2_ allyl group) corresponded to the DS.

Several molar ratios between the reagents (mol_allyl bromide_/mol_saccharide_) were tested, obtaining DS values that increased as the molar ratio increased. In the end, molar ratios of 6 and 3 were chosen as optimal values for the functionalization of the α,α′-trehalose and of the MG, respectively, because they allowed an average DS per molecule between 1 and 2 to be obtained. The use of monomers with a low DS was a way to reduce the possibility of cross-linking during the following copolymerization reactions and to favour the formation of linear chains.

### 3.2. Synthesis and characterization of the vinyl acetate copolymers

**Figure 2.**
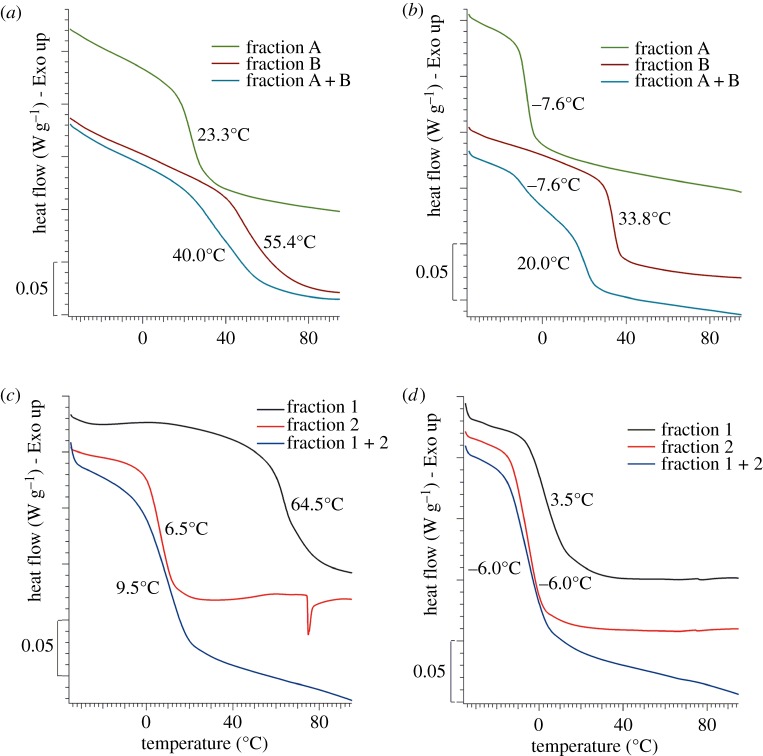
DSC analysis (second heating scan): (*a*) ATR/VAc copolymer, (*b*) AMG/VAc copolymer, (*c*) ATR/VOH copolymer and (*d*) AMG/VOH copolymer.

**Figure 3.**
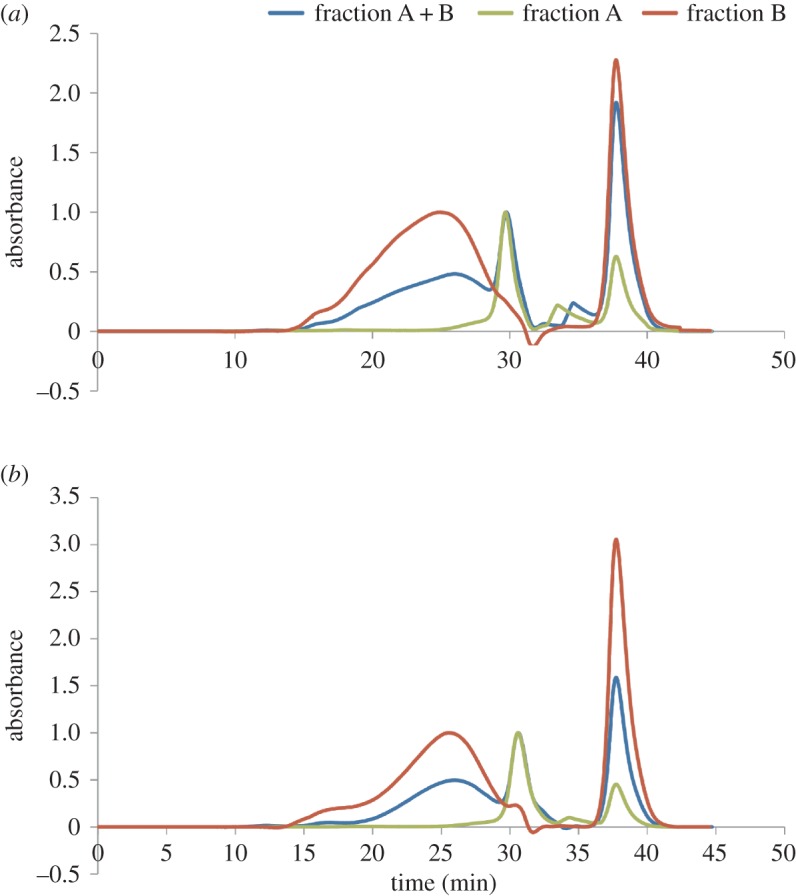
SEC analysis of the ATR/VAc copolymer (*a*) and of the AMG/VAc copolymer (*b*): fraction A + B blue line, fraction A green line, fraction B red line.

**Scheme 2.**
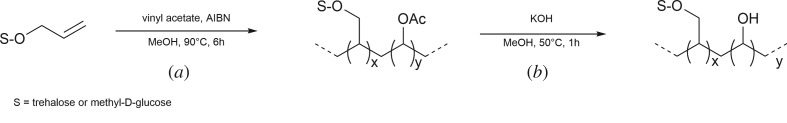
Synthesis of the vinyl acetate copolymers (*a*) and of the vinyl alcohol copolymers (*b*).

The syntheses of two different vinyl acetate copolymers, respectively using ATR or AMG as comonomers, were performed in methanol (scheme 2*a*), even if water is commonly used as the dispersion medium for the homo- and copolymerization of the vinyl acetate. The synthesis with ATR was also performed using ethanol as the best solvent for green chemistry obtaining comparable results in terms of yield and structural characteristics. In this way, using a low molecular alcohol (methanol or ethanol), it was possible to obtain pure copolymers and to characterize them, avoiding the presence of the additives, which are essential for their synthesis in water dispersion (i.e. protective colloid, surfactants, buffers). Methanol was used as preferred solvent in all the syntheses for a better work-up for the subsequent spectroscopic characterization. The reactions were performed using AIBN as a radical initiator in order to compare these results with analogous syntheses of the polyvinyl acetate homopolymer and other copolymers [54]. The conversions in the presence of ATR (65–62%) and AMG (57%) were lower than that obtained in similar reaction conditions for polyvinyl acetate homopolymer (75%). Nevertheless, the presence of the allyl monomers does not drastically reduce the yield, because the obtained values were comparable to those of other vinyl copolymers (70–73%) [54]. All reactions were performed under nitrogen atmosphere in order to exclude oxygen, which acts as an inhibitor of the free radical polymerizations and as an oxidizing agent for the allyl and vinyl groups. The work-up procedure was the same for both the reactions. After 6 h at 90°C, the solvent and the residual vinyl acetate were removed by distillation at reduced pressure. Then, an extraction in acetone was performed to purify the products from the salts and the unreacted allyl saccharides. The copolymers were extracted in acetone and identified as fraction A + B for both the reactions. Those fractions contained chains of allyl saccharide/vinyl acetate copolymer with different ratios between the units of the two comonomers, probably together with chains of vinyl acetate homopolymer. NMR and FT-IR spectra of the fractions A + B of both copolymers are reported in the electronic supplementary material, figures S4–S6. The characteristic signals of the vinyl acetate units were visible in the NMR spectra of both copolymers at similar chemical shifts. In particular, in the ^1^H-NMR spectra the signals were present at about 1.84 ppm (CH_3_–CO–CH–C**H_2_**–), 2.00 ppm (C**H_3_**–CO–CH–CH_2_–) and 4.90 ppm (CH_3_–CO–C**H**–CH_2_–) and in the ^13^C-NMR spectra at about 20.0 ppm (**C**H_3_–CO–CH–CH_2_–), 40.0 ppm (CH_3_–CO–CH–**C**H_2_–), 68.0 ppm (CH_3_–CO–**C**H–CH_2_–) and 172.0 ppm (CH_3_–**C**O–CH–CH_2_–). In the NMR spectra of both the fractions A + B, the signals of the allyl saccharides structure were also observable. It is worth noting that the characteristic signals of the allyl groups were still visible, even if their intensities were lower compared to the spectra of the starting ATR or AMG monomers. This result was due to the fact that the DS of the monomers was evaluated as an average value and, therefore, a DS value between 1 and 2 was in agreement with the presence of some ATR or AMG molecules with more than one functionalizing group on them. Furthermore, allyl groups had not reacted completely during the copolymerizations, their reactivity being lower than that of the vinyl groups. On the other hand, it was possible to exclude the presence of unreacted free monomers based on the applied work-up procedure because of their insolubility in acetone. Consequently, the signals of unreacted allyl groups which are visible in the spectra were attributed to the monomer units present as side groups in the copolymer chains. The FT-IR spectra of both the fractions A + B showed the characteristic bands of the acetate group at 1736 cm^−1^ (C=O stretching), 1430 and 1373 cm^−1^ (CH_3_– δ acetate group), together with the characteristic bands of the allyl saccharides structures. The fractions A + B of the ATR/VAc and the AMG/VAc copolymers were also characterized by DSC and SEC analyses. DSC thermograms (figure 2*a*,*b*, blue curves) showed the presence of a *T*_g_ at about 42°C for the ATR/VAc copolymer and at about 25–28°C for the AMG/VAc copolymer. The SEC analyses (figure 3, blue curve) showed a very large peak centred at about 25 min and a narrow peak at about 30 min as regards the ATR/VAc copolymer, while in the case of AMG/VAc copolymer the peaks had a slightly higher retention time. The first peak was related to copolymer chains with a behaviour similar to that of a polyvinyl acetate homopolymer, obtained as a standard reference by applying the same procedure used for the synthesis of the copolymers (electronic supplementary material, figure S7). However, it is worth noting that in the case of ATR/VAc and AMG/VAc copolymers there was a significant presence of species having high hydrodynamic volume that were not present in the polyvinyl acetate homopolymer. The second peak was sharper than the other and it was attributed to copolymer chains with lower molecular weights, because its retention time was too low to correspond to one of the starting monomers.

**Figure 4.**
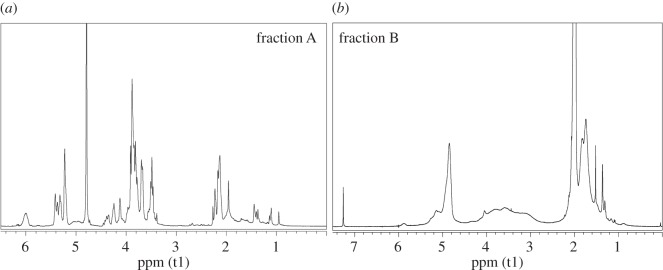
^1^H-NMR spectra of fraction A (*a*) and fraction B (*b*) of the ATR/VAc copolymer.

**Figure 5.**
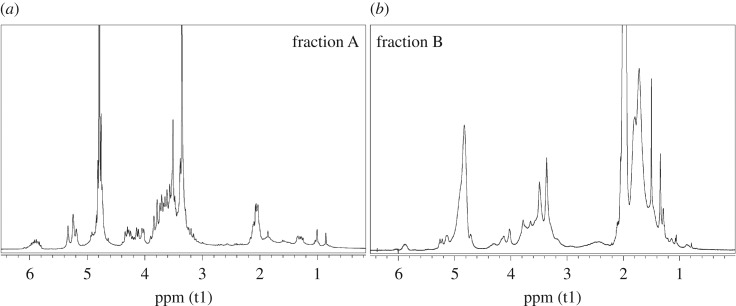
^1^H-NMR spectra of fraction A (*a*) and fraction B (*b*) of the AMG/VAc copolymer.

**Figure 6.**
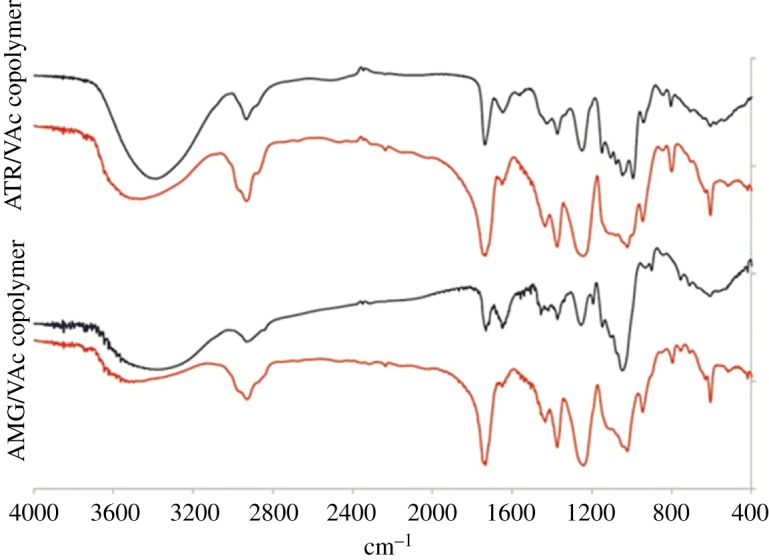
FT-IR spectra of the ATR/VAc and AMG/VAc copolymer (fraction A black line, fraction B red line).

To better characterize the products which gave the two different peaks in the SEC analyses, an extraction in water of the fractions A + B of the ATR/VAc and AMG/VAc copolymers was performed and two fractions with different solubility were obtained for each copolymer: fraction A (soluble in water) and fraction B (insoluble in water). All fractions A and fractions B were characterized by NMR spectroscopy (figures 4 and 5), FT-IR spectroscopy (figure 6), DSC (figure 2*a*,*b*) and SEC analysis (figure 3 and table 4). Both fractions A contained copolymer chains rich in saccharide units, as demonstrated by their FT-IR and ^1^H-NMR spectra, in which the intensity of the characteristic signals of the allyl saccharide units was higher than that of the signals of the vinyl acetate units. The presence of a high amount of allyl saccharide units also justified the results of the SEC analyses performed on both fractions A, which showed only the narrow peak at about 30–31 min, that corresponded to low molecular weights. As expected, fraction A of ATR/VAc had a higher hydrodynamic volume, i.e. higher molecular weight, than fraction A of AMG/VAc, since AMG is smaller than ATR. In both cases, the less reactive allyl groups may have influenced the copolymerization, contributing to stop the growth of the chains. The high amount of allyl saccharide units was also in agreement with the water solubility of these fractions.

**Table 4.** Results of SEC analyses on vinyl acetate copolymers.

		*M*_n_ (g/mol)	*M*_w_ (g/mol)	*M*_p_ (g/mol)	*Ð*
ATR/VAc copolymer	fraction A + B	4800	64 200	5900	13.40
	fraction A	6000	6800	6000	1.14
	fraction B	19 300	102 200	26 900	5.28
AMG/VAc copolymer	fraction A + B	8200	97 900	4300	11.97
	fraction A	2400	4300	4400	1.79
	fraction B	20 600	103 700	21 900	5.03

The *T*_g_ values recorded for fractions A (figure 2*a*,*b*, green curves) of both the copolymers were approximately 23°C and −8°C for ATR/VAc and AMG/VAc copolymers, respectively. On the contrary, the copolymer chains in both fractions B were rich in vinyl acetate units, as confirmed by the higher intensity of their characteristic signals in the FT-IR and ^1^H-NMR spectra compared to those of the allyl saccharide units. The presence of a high amount of vinyl acetate units justified the insolubility in water and the higher molecular weights of both the fractions B, because the more reactive vinyl groups may have favoured the formation of longer chains. The DSC thermograms of these fractions B (figure 2*a*,*b*, red curves) showed that the *T*_g_ of the ATR/VAc polymer is around 55°C, and that of the AMG/VAc polymer is around 34°C. Interestingly, the thermogram of the fractions A + B of both the copolymers displayed a *T*_g_ at a value intermediate between those of fraction A and B (40°C in ATR/VAc copolymer and 20°C in AMG/VAc copolymer), indicating a strong interaction between the two fractions A and B. In the case of AMG/VAc, however, the *T*_g_ at −8°C was still visible, suggesting that a part of the fraction A remained separated from the mix.

### 3.3. Synthesis and characterization of the vinyl alcohol copolymers

Finally, in order to achieve totally water-soluble products, the ATR/VAc and the AMG/VAc copolymers were hydrolysed to the corresponding vinyl alcohol copolymers (scheme 2*b*). In this way it was also possible to prevent the hydrolysis of the acetate groups from occurring after the application on the work of art, if the vinyl acetate copolymers were used to treat the degraded material. In fact, the hydrolysis of acetate groups produces acetic acid and can cause a hazardous decrease of the pH which, paradoxically, can accelerate the degradation of the artefact, achieving an effect that is the opposite to the desired one.

**Figure 7.**
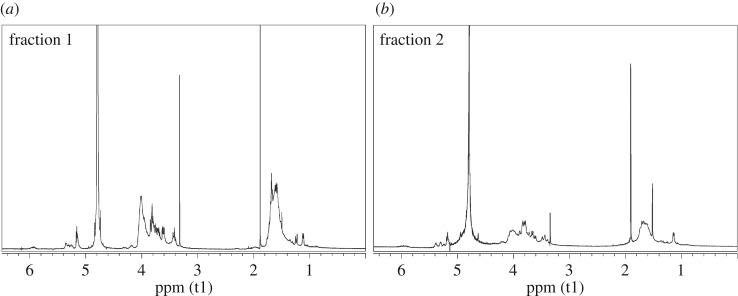
^1^H-NMR spectra of the fraction 1 (*a*) and fraction 2 (*b*) of the ATR/VOH copolymer.

**Figure 8.**
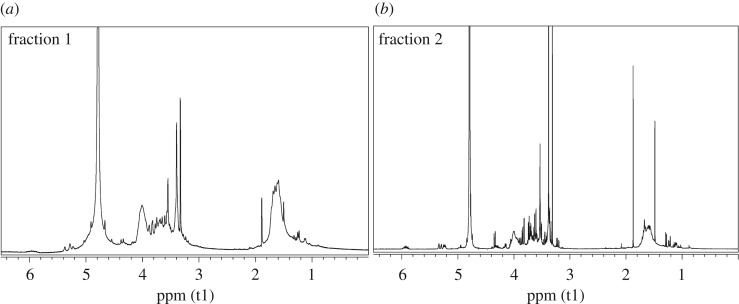
^1^H-NMR spectra of the fraction 1 (*a*) and fraction 2 (*b*) of the AMG/VOH copolymer.

**Figure 9.**
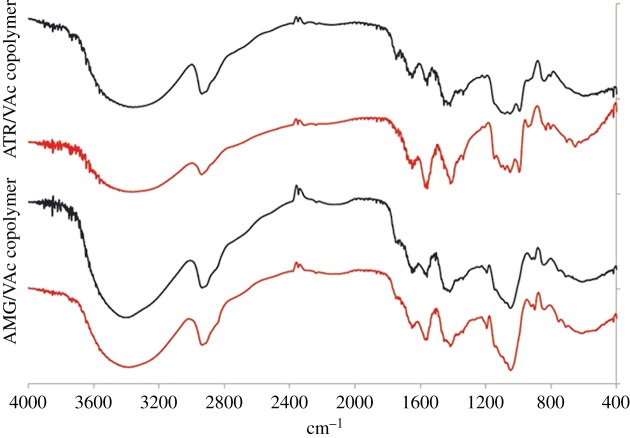
FT-IR spectra of the ATR/VOH and AMG/VOH copolymer (fraction 1 black line, fraction 2 red line).

The reactions were performed following a standard procedure [55], that is a transesterification in methanol in the presence of a catalytic amount of potassium methoxide. The fractions A + B of both vinyl acetate copolymers were used as starting material, and at the end of each hydrolysis two fractions with different solubility in methanol were obtained. In particular, the fraction 1, insoluble in the reaction mixture, was separated by centrifugation from the fraction 2, soluble in the reaction mixture, which was recovered by distilling the solvent at reduced pressure. The characterization of the two separate fractions obtained from the hydrolysis of each copolymer was performed by NMR spectroscopy, FT-IR spectroscopy and by DSC analysis. In the ^1^H-NMR and ^13^C-NMR spectra (figures 7 and 8), the characteristic signals of the vinyl alcohol units were visible at about 1.60 and 4.00 ppm, and at about 44.0 and 66.0 ppm, respectively. Comparing the ^1^H-NMR spectra of the fractions 1 with those of fractions 2, the ratio between the intensities of the characteristic signals of the vinyl alcohol units and those of the characteristic signals of the saccharide units was different. In particular, the copolymer chains in fractions 1 of both the copolymers were richer in vinyl alcohol units than those in fractions 2. This feature was consistent with the different solubility in methanol of the two fractions and with the fact that the starting fractions A + B of the vinyl acetate copolymers contained chains rich in vinyl acetate units and chains rich in allyl saccharide units. Concerning the degree of hydrolysis, the characteristic bands of the acetate group, in particular the C=O stretching at 1747 cm^−1^, were still visible in the FT-IR spectra of fractions 1 and 2 of both the copolymers (figure 9), even if they were less intense than in the spectra of the starting fractions A + B of the vinyl acetate copolymers. Unfortunately, it was not possible to evaluate the degree of hydrolysis with the general procedure, that consists of using the integral values of the ^1^H-NMR signals of vinyl acetate (2.00 ppm, CH_3_–CO) and of vinyl alcohol (4.00 ppm, CH_2_–CH(OH)–) units, due to the overlapping of the latter signal with those of the saccharide structures. Nevertheless, the signals of the vinyl acetate units had very low intensities in all the NMR spectra so it was possible to conclude that the hydrolysis was almost complete. DSC analyses were performed on fractions 1 and 2 of both copolymers and also on fractions 1 + 2 (figure 2*c*,*d*). Those latter fractions were obtained by simply mixing fractions 1 and fractions 2 and they were analysed to evaluate their thermal behaviour in view of their possible application as mixtures. The presence of the allyl saccharide comonomer units contributed to decrease of the *T*_g_ values of all fractions with respect to that reported in the literature for the polyvinyl alcohol homopolymer (i.e. 85°C [56]). Nevertheless, this decrease was more evident for fractions 2, which were richer in allyl saccharide units with respect to fractions 1. As in the case of the vinyl acetate copolymers, the fractions 1 + 2 showed a single value of *T*_g_ and this behaviour indicated a strong interaction between the copolymer chains inside these fractions.

## 4 Conclusion

New synthetic biopolymers were synthesized starting from renewable resources, as α,α′-trehalose and d-glucose, obtaining suitable products for applications like the adhesion or the consolidation of degraded works of art. In particular, the use of saccharides allowed products to be obtained that show high affinity and compatibility for the cellulosic substrates, like paper or wood.

Allyl saccharide monomers were synthesized in water using allyl bromide as a functionalizing agent in order to introduce a reactive group in the structure of the saccharides. The molar ratio between the reagents (mol_allyl bromide_/mol_saccharide_) was chosen in order to have an average DS value of 1–2 on the final molecules. Considering the low reactivity of the allyl group, the choice of limiting the functionalization of the monomers allowed vinyl acetate copolymers with low molecular weights to be obtained, simultaneously decreasing the possibility of a cross-linking during the copolymerization. These molecular weights are not only capable of ensuring good mechanical properties for the polymers, but also facilitate their penetration into porous materials, like wood or paper.

The syntheses of the vinyl acetate copolymers were performed using methanol or ethanol as a solvent in order to obtain pure products avoiding the presence of additives. NMR, SEC and DSC characterization underlined the presence of copolymer chains characterized by different solubility in water, which was related to their different composition in terms of ratio between the units of the comonomers.

Finally, vinyl alcohol copolymers were obtained by hydrolysis of the corresponding vinyl acetate copolymers, with the aim of obtaining water-soluble products suitable for the treatment of wood and paper. Applicative studies on the treatment of the archaeological waterlogged wood with the newly synthesized biopolymers are in progress in our laboratory and the preliminary results are showing interesting behaviour (S Dominici, A Papacchini, G Di Giulio, M Fioravanti, A Salvini, unpublished data).

## Supplementary Material

Supporting information: The 1H-NMR, 13C-NMR and FT-IR spectra of ATR, AMG, ATR/VAc copolymer (fraction A+B) and AMG/VAc copolymer (fraction A+B) and the SEC analysis of the vinyl acetate homopolymer

## Data accessibility

The ^1^H-NMR, ^13^C-NMR and FT-IR spectra of ATR, AMG, ATR/VAc copolymer (fraction A + B) and AMG/VAc copolymer (fraction A + B) and the SEC analysis of the vinyl acetate homopolymer referenced in the text are included in the electronic supplementary information.
