# Supplementary material for: Modified α,α′-trehalose and d-glucose: green monomers for the synthesis of vinyl copolymers
Source: R Soc Open Sci. 2018 May 23;5(5):171313. doi: 10.1098/rsos.171313 (PMC5990774; doi:10.1098/rsos.171313)
Supplement: Supporting information: The 1H-NMR, 13C-NMR and FT-IR spectra of ATR, AMG, ATR/VAc copolymer (fraction A+B) and AMG/VAc copolymer (fraction A+B) and the SEC analysis of the vinyl acetate homopolymer [file rsos171313supp1.docx]

Electronic supplementary information


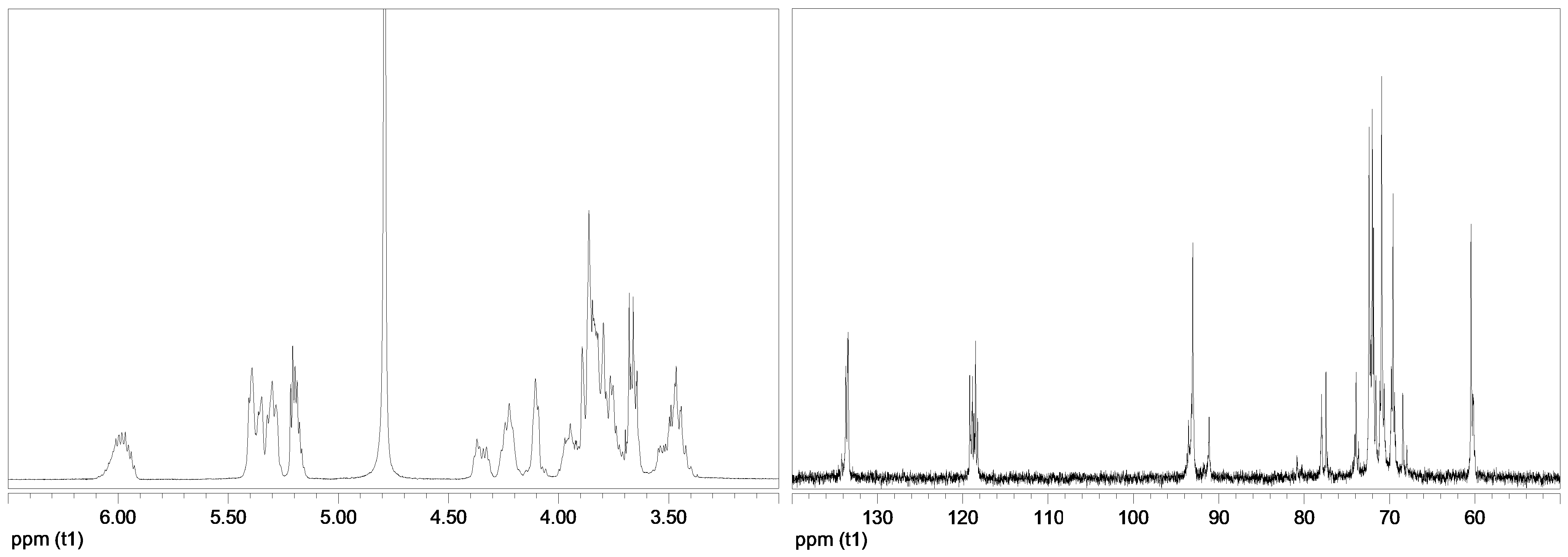


**S1** ^1^H-NMR and ^13^C-NMR spectra of the allyl α,α’-trehalose (ATR)


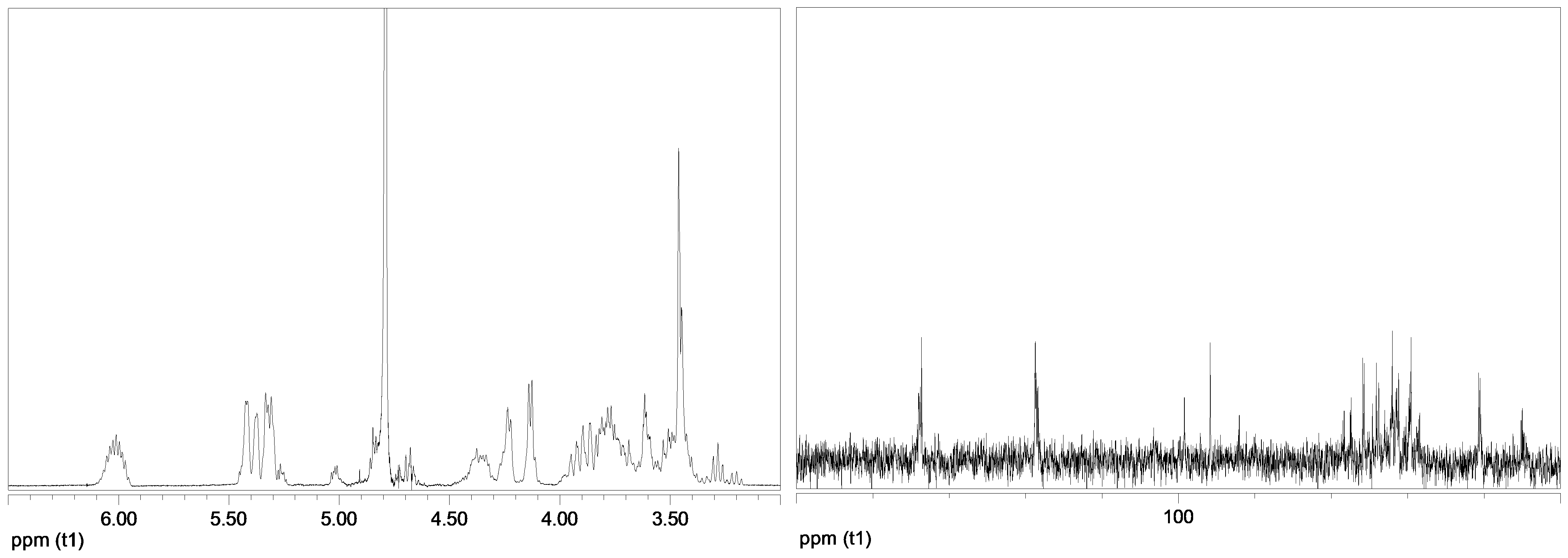


**S2** ^1^H-NMR and ^13^C-NMR spectra of the allyl methyl D-glucopyranoside (AMG)


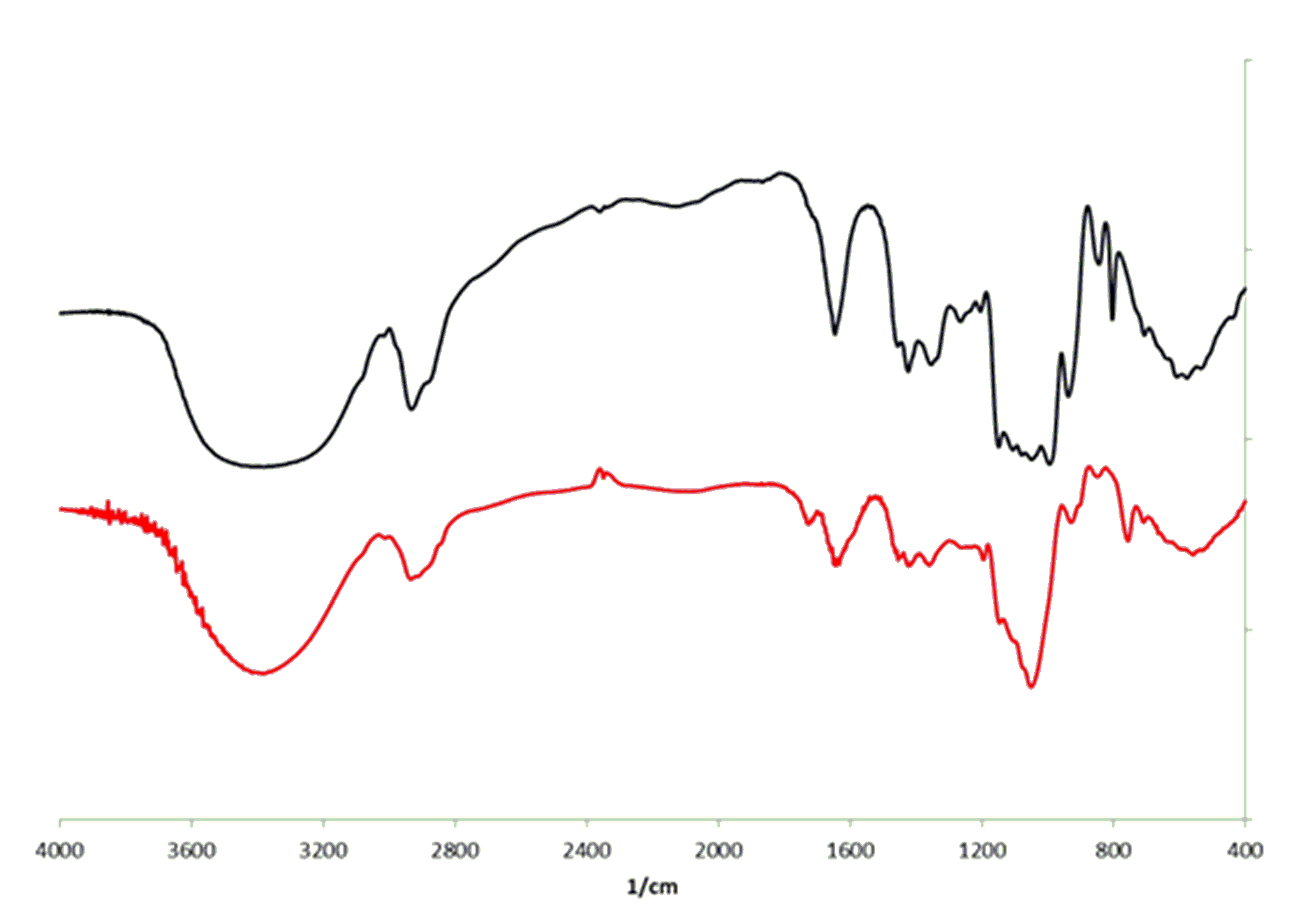


**S3** FT-IR spectra of the ATR (black line) and of the AMG (red line)


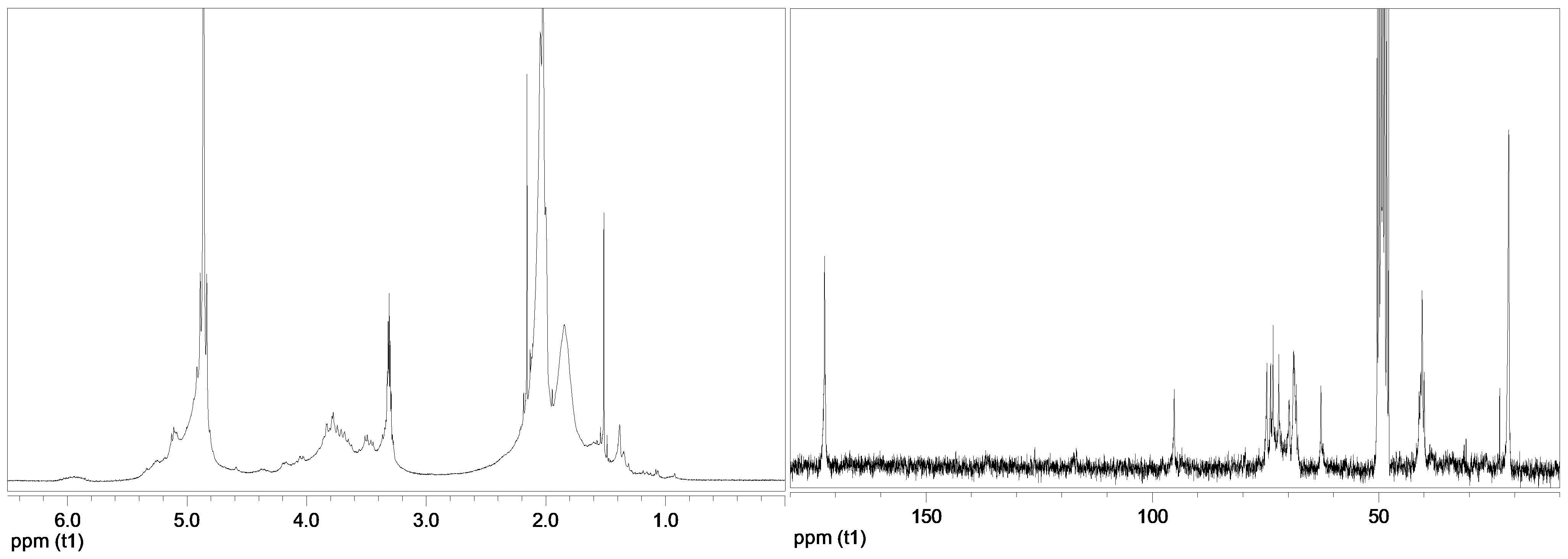


**S4** ^1^H-NMR and ^13^C-NMR spectra of the allyl α,α’-trehalose/vinyl acetate (ATR/VAc) copolymer (fraction A+B)


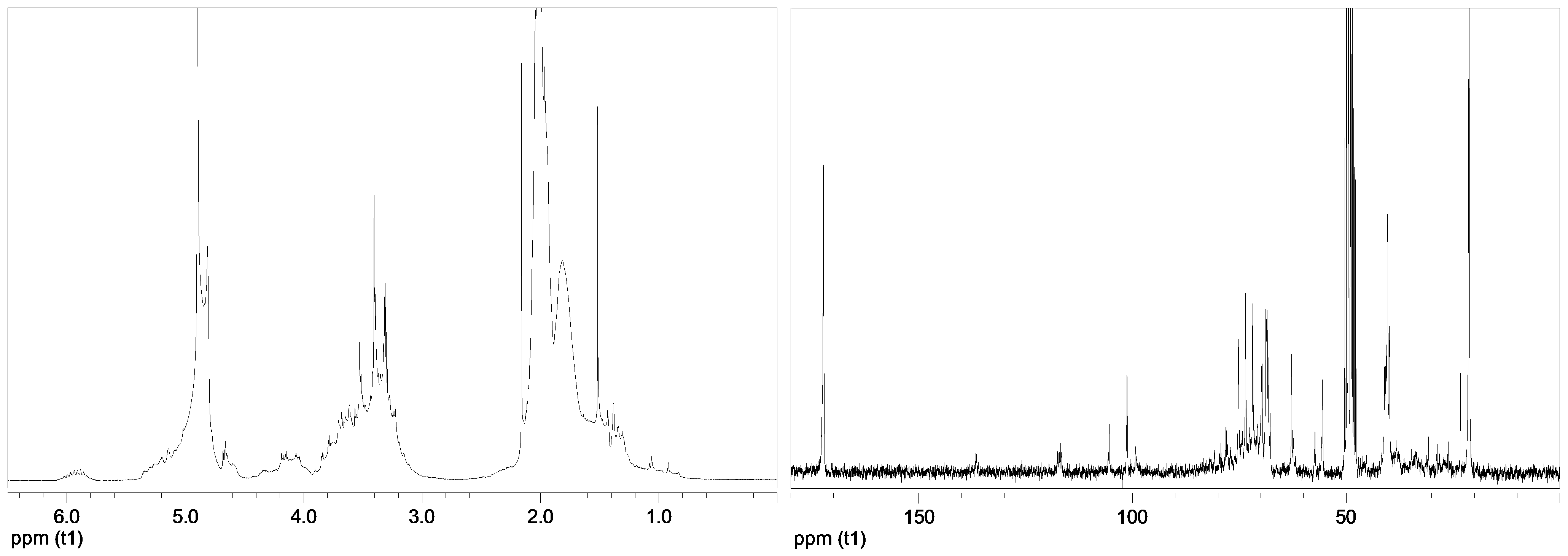


**S5** ^1^H-NMR and ^13^C-NMR spectra of the allyl methyl D-glucopyranoside/vinyl acetate (AMG/VAc) copolymer (fraction A+B)


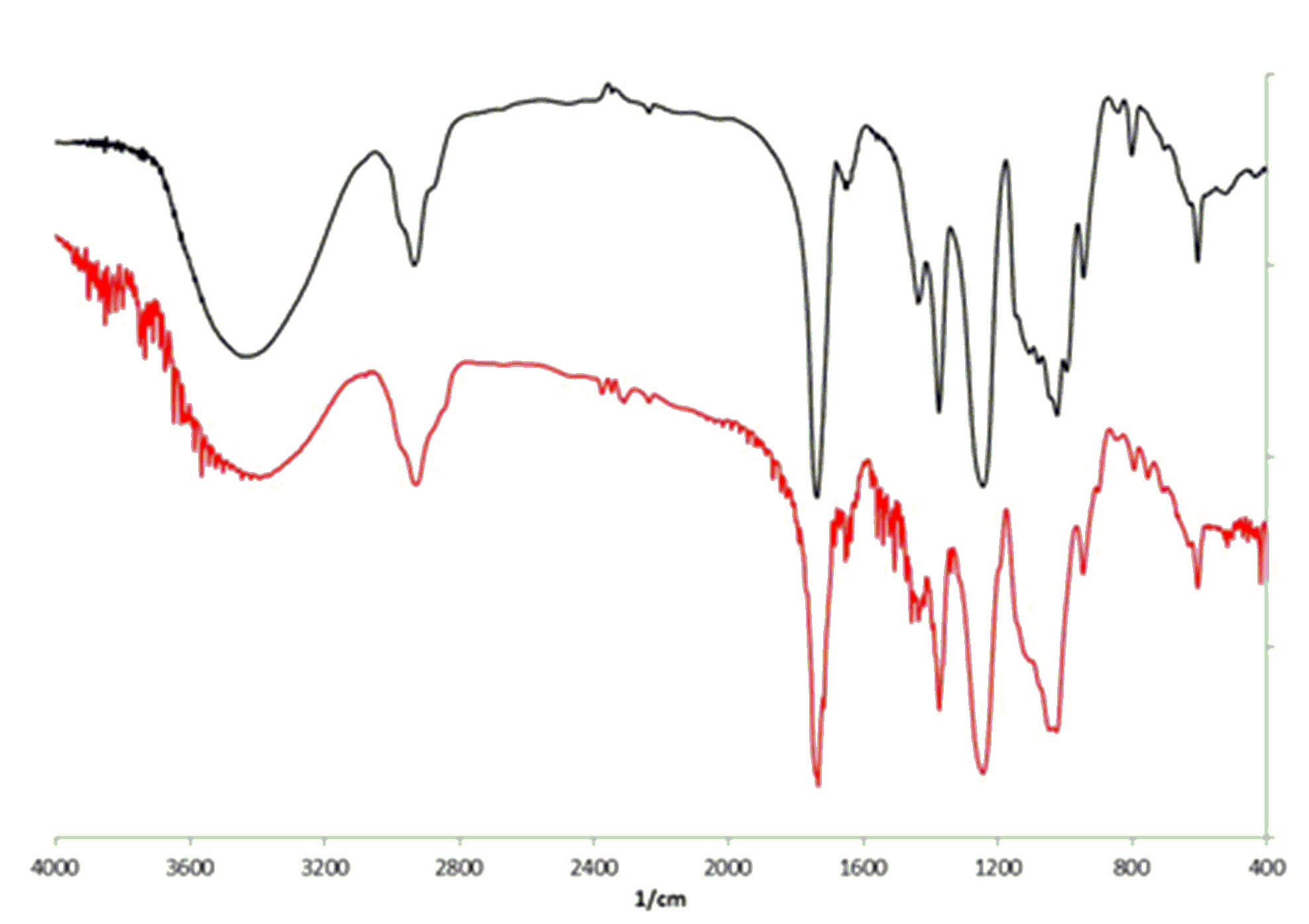


**S6** FT-IR spectra of the fraction A+B of the ATR/VAc copolymer (black line) and of the AMG/VAc copolymer (red line)


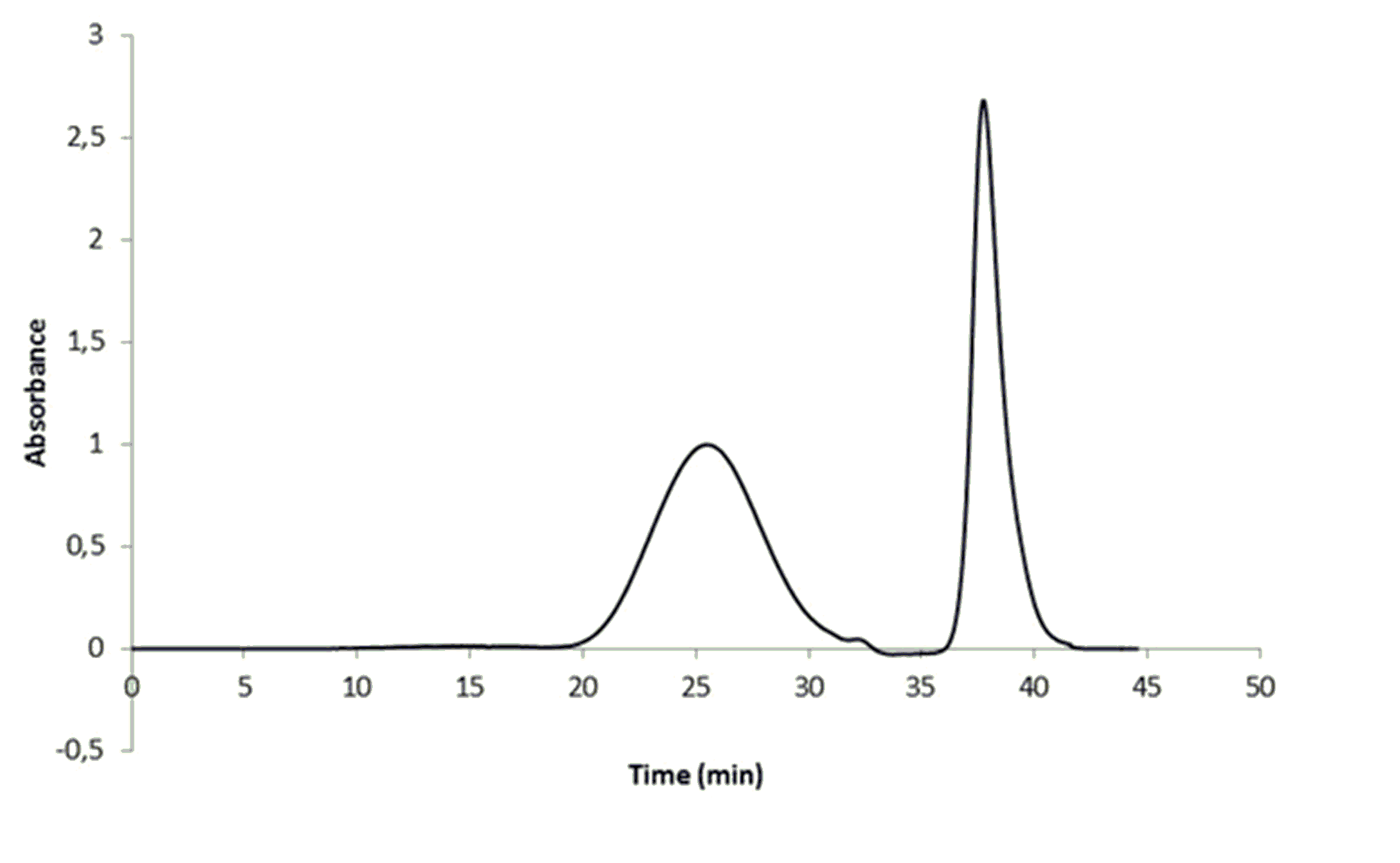


**S7** SEC analysis of the vinyl acetate homopolymer
